# Actions speak louder than words: modifications of the applied academic books and their reflections on students’ academic success, academic enjoyment, and academic resilience

**DOI:** 10.1186/s40359-024-01650-8

**Published:** 2024-03-15

**Authors:** Wael Matar Hasan Alharbi

**Affiliations:** Department of Arabic Language, College of Education in Al-kharj, Prince Sattam bin Abdulaziz University, Al-kharj, Saudi Arabia

**Keywords:** Modifications of the applied academic books, Academic success, Academic enjoyment, Academic resilience, EFL learners

## Abstract

Innovation is the process of renewing and updating a domain, a product, or a service by introducing new methods, introducing new techniques, or generating effective concepts to produce new value. Considering universal as well as local books, a teacher may find some sections that need modifications and supportive sections. These changes may have an impact on language learners’ emotional and/or cognitive development. The purpose of this research was to shed light on the use of creative activities and their consequences on academic enjoyment, academic resilience, and academic performance. To that purpose, two groups of EFL (English as a foreign language) learners from Saudi Arabia (public schools) participated in this research and were requested to take pre-and post-tests to assess their level of academic engagement, academic resilience, and academic performance in language skills. According to the data analysis via the Multivariate Analysis of Variance (MANOVA), the experimental group outperformed their counterparts in the control group in terms of academic enjoyment, resilience, and academic performance due to considered modifications in the applied academic books. The study’s pedagogical ramifications are highlighted in the conclusion.

**Supplementary Information:**

The online version contains supplementary material available at 10.1186/s40359-024-01650-8.

## Introduction

It is widely acknowledged that textbooks are the most important component of language education [1]. They are referred to as the visual heart of any English Language Teaching (ELT) program [2]. Within the context of the teaching and learning process, coursebooks assume the role of the principal repository of information. In addition, teachers make use of them to achieve their pedagogical goals and improve the overall learning experience for their pupils [3]. According to [4], it is a very rare occurrence for teachers to be able to effectively handle teaching without relying on a textbook.

Textbook review and analysis are essential for assessing the appropriateness of textbooks for an English program or classroom and making necessary adjustments. Following [5], textbook evaluation is a practical linguistic activity that enables teachers, supervisors, administrators, and others who create materials to evaluate the influence that the materials have on the people who use them. The significance of the function that textbook analysis and assessment play in the process of teaching English as a foreign language has been underlined by some studies [6, 7]. Advocates of CLT support an educational approach that prioritizes the cultivation of skills, exploration of information, and cooperation among students [8]. Language acquisition occurs inside intimate classroom environments via activities that include collaboration in both group and couple settings [9].

Using a qualitative study approach, [10] looked at the challenges, restrictions, and issues that English teachers in Iran encountered when using the Prospect Series. According to their study, some instructors expressed concerns about the lack of facilities and a stimulating learning environment in impoverished regions. They believe that addressing this problem is crucial to effectively use the newly released series. Within the scope of their investigation, [11] used a detailed checklist to evaluate the Prospect series in Iran, with a particular emphasis on the instructor’s perspectives. The findings of this study indicated that articulation strategies, language activities, and activities must be improved and modified to meet the requirements of the research. In addition, teachers believed that the vocabulary section of Helen Stephenson’s book “Life” was the most beneficial aspect of the book.

## Literature review

The ability to successfully adjust oneself to tough or terrifying circumstances, as well as the ability to properly handle routine setbacks and challenges, are both necessary components for developing resilience [12]. Academic Resilience (AR) is a complicated term, and the formation and progression of AR are influenced by some elements that play a significant role [13]. These qualities include personality, temperament, as well as distinctive talents such as active problem-solving and psychological traits. The AR program instills students with the confidence necessary to take chances, hence reducing the worry they have about academic failure or dropping out of school [14]. In the words of [15], resilience may be defined as the ability to sustain regular development and achieve desirable changes even in the face of significant adversity. Furthermore, [16] provided evidence that demonstrates the significant impact of reflecting on past events and seeking help on the progress of AR. This data was presented within the framework of the progression of AR. As defined by [17, 18], AR refers to the characteristics that differentiate those who achieve success from those who do not.

Following the findings of [19], cultivating resilience may be a crucial aspect of students’ well-being and engagement in second language education [20]. also assert that resilience is potentially valuable and plays a significant role in achieving success in acquiring a new language. Therefore, it is crucial to further investigate the attributes of resilience in language learners and how it relates to the process of language acquisition. Similarly, [21] showed that self-efficacy, L2 grit, and AR had a favorable impact on the performance of EFL students in online assessments. Additionally, they determined that these characteristics may also aid in preventing demotivation and disengagement in the process of language acquisition.

Previous research has demonstrated that students who are learning a language and have a high degree of resilience have a larger feeling of competition within the classroom setting. On the other hand, pupils who lacked resilience had lower levels of attention and assessed the difficulty of their reading courses as higher [22]. Within the framework of language acquisition, the idea of buoyancy, which is a similar construct to resilience, was studied in the study that was carried out by [19]. Through their research, they discovered that self-efficacy and self-regulation were powerful predictors of L2 buoyancy. In addition to this, they found that buoyancy was a significant factor in determining the success of L2 students. Some studies that have been conducted on the topic of resilience in language acquisition have shown that it has a substantial and positive link with approach coping and pleasure coping while displaying a negative correlation with avoidant coping [23, 24].

It has been shown in previous studies [22–25] that resilience might be a strong predictor of patience in second language acquisition. Research conducted in the past has shown that several characteristics, including demotivation, self-efficacy, ideal L2 self, and self-regulation, strongly predicted L2 resilience or its correlated dimensions (i.e., buoyancy) [16]. This study was undertaken to determine whether or not linguistic mindsets, prospective selves in the second language, and predicted communication capacity are capable of predicting resilience among those who are learning English as a foreign language.

In the field of positive psychology, enjoyment is characterized as the emotion evoked by the realization of a goal [26]. In the work of [27], enjoyment may be experienced on several levels, including emotional, intellectual, motivational, communicative, and physical. Students’ good feelings as a result of rewarding language learning experiences constitute the emotional component of pleasure. Constructive evaluation of language learning experiences is defined by the intellectual component. In line with [28, 29], the satisfaction that students have affects their motivation to study, their social connections with teachers and classmates, as well as their physical health. Academic Enjoyment (AE) is inherently a fluid concept. According to [27], linguistic AE evolves over time as a result of individual differences in personality and classroom context. The study conducted by [30, 31] demonstrates that interactions between teachers and students are essential to the development of AE in the classroom. In addition, achieving a balance between the traits that are context-oriented and the mental needs of the learners is another way to inspire happiness in the classroom [32].

The control-value hypothesis of accomplishment emotions posits that students’ judgments of their own control and value systems serve as the immediate precursors of achievement emotions, which impact their subsequent academic performance [33]. Using control-value theory as a foundation, researchers found that factors such as mental capacities, accomplishment objectives, psychological engagement, and strategy utilization would mediate the relationship between accomplishment emotions and educational achievement [34]. Academic self-concept could also mediate the relationship between achievement emotions and academic performance, as shown by the symbiotic relationships between the two in control-value theory and empirical evidence [35].

Emotions linked to doing an action or seeing the outcome of an action are known as achievement emotions. In addition, as noted by [34, 35], sentiments of achievement are more particular to certain domains. Concerning research by [26], it is essential to first investigate the problem at hand and the surrounding circumstances before addressing a particular sense of success. The idea that students’ self-discipline, learning approaches, motivation, and the activation of cognitive resources are all positively connected with pleasant emotions, such as pleasure, and that each of these emotions plays a crucial role in the process of acquiring information is generally acknowledged [36].

## Aims and hypotheses of the Present Study

This study was carried out to examine the impacts of alterations in applied materials and the repercussions that these modifications have on the academic achievement, academic pleasure, and resilience of students who are learning English as a foreign language. At every level of education in Saudi Arabia, beginning with the first grade of secondary school and continuing up to the doctoral level, students are required to take English classes. A strong command of the English language is essential for advancing one’s academic career as well as one’s professional career. Research efforts have focused on the importance of CLT in EFL. However, further study is required to better explore the impact of positive accomplishment emotions, such as pleasure, on academic achievement in the EFL environment. This research might provide valuable insights into providing innovations in-class activities and their reflections on students’ academic success, academic enjoyment, and resilience. The current research addressed the gap in knowledge by investigating the consequences of modifications of existing exercises in applied materials on academic success, AE, and AR among secondary school students studying English as a foreign language. The study investigated three hypotheses:

### Hypothesis 1

Modifications of the applied academic books foster academic success in an EFL setting.

### Hypothesis 2

Modifications of the applied academic books foster academic enjoyment in an EFL setting.

### Hypothesis 3

Modifications of the applied academic books foster academic resilience in an EFL setting.

### Research design

#### Participants

The participants in this research study were comprised of a group of 85 individuals who had taken part in EFL training at Prince Sattam bin Abdulaziz University, Saudi Arabia. To select language learners to participate in the program, the results of the Oxford Quick Placement Test were used. There were no female students in the current research; all of the students were male attending high schools in Saudi Arabia and varied in age from 16 to 17 years old. Within the span of a single academic year, it was predicted that they would be able to successfully complete the book “Life” written by Helen Stephenson. Individuals who took part in this inquiry offered their informed consent to participate in the investigation in a way that was both spontaneous and voluntary. Participants in this investigation gave their informed consent.

### Measures

To evaluate the student’s English proficiency, the Oxford Quick Placement Test (OQPT) (See Additional File 1) was administered. Students who obtained scores within the interval of 0.4 and 0.6 on this English language assessment, with a range of prospective values from 0.1 to 0.9, are deemed to have attained English language proficiency at the intermediate level. The reliability of the OQPT was assessed using Cronbach’s alpha, which yielded favorable results with a value of 0.91.

Before and following the treatment, the participants completed a researcher-created examination on the subject matter to gauge their language development. The examination comprised a total of 50 inquiries, with 12 queries allocated to each of the following four sections: speaking (10 queries), listening (10 queries), writing (15 queries), and reading (15 queries). Two psychometricians and two EFL instructors evaluated the content and face validity of the examination; the results of this evaluation informed modifications to the instrument. A test-retest reliability analysis was subsequently conducted on a sample of 28 EFL learners who possessed English proficiency at the intermediate level. To ascertain the durability of the results, the identical test was re-administered to the individual several weeks later. The obtained Pearson’s R-value (*r* =.873, *p* <.05) proved to be highly valuable.

The Foreign Language Enjoyment Scale (FLES), which was created and verified by [26], was used to investigate the level of enjoyment experienced by language students. There are twenty-one items on the FLES, each of which is rated on a Likert scale from one to five, with the highest possible score being “strongly disagree” and the lowest possible score being “strongly agree.” The reliability of the FLES in this investigation was determined to be adequate, as shown by the report of Cronbach’s alpha, which was calculated to be 0.867.

For the AR evaluation, the Academic Resilience Scale (ARS), which was devised by [14], was utilized. The Likert scale comprises a total of twenty-six distinct items, with Likert values ranging from one to five for each item. The remaining items can be classified into the following five categories: self-regulation (2 items), subjective pleasure (9 items), empathy (7 items), and sociability (3 items). The results of this study suggested that the ARS was reliable, as its value lagged between 0.831 and 0.902, which is within the acceptable range.

### Data Collection procedures and Analysis

Immediately after the pre-test, one of the researchers, who also acted as the language instructor for all of the sessions that were attended by either the EG or CG participants, gave instructions to the participants. The instruction that was provided to the students who were assigned to the CG was the normal instruction, and there were no modifications or supplemental material utilized in their instruction beyond the basic textbook. The instruction provided for the EG was designed to enhance the learners’ language skills. Besides the exercises defined in their books, some supplementary tasks were developed to enhance language learning. Some of the tasks were also modified. All changes and modifications were based on the need analysis and multiple intelligences of the students and the possible shortcomings of the applied books. After this project and after the instruction had been completed, the post-test was given to the students. The exam was designed to assess the achievements of the students as well as their AR and AE in both the CG and the EG, and it was also intended to identify the degree to which the program had been effective. The language of the questionnaires was English as the students were qualified enough to understand and answer them. It should be noted that mid-term and final exams for both CG as well as EG were the same. After that, MANOVA was carried out to investigate the tracks of innovations in-class activities and their reflections on students’ academic success, academic enjoyment, and resilience.

## Results

Using parametric or no-parametric statistical tests is required to ensure normality distribution. The researchers first ran a Kolmogorov-Smirnov test to delve into the state of data distribution and the results showed the data were normal as the p values were higher than 0.05. Thus, running independent samples T-test and MANOVA was safe for analyzing the data. At first, the researchers checked the pretests of the participants through an independent samples T-test to check if the groups were homogenous before the treatment.

**Table 1:** Descriptive statistics (Pretests of AS, AE, and R)

	Groups	N	Mean	Std. Deviation	Std. Error Mean
SAS Pre	EG	40	35.2500	22.00320	3.47901
	CG	39	29.1282	8.54456	1.36822
R Pre	EG	40	105.6250	22.82451	3.60887
	CG	39	113.2051	25.15549	4.02810
AE Pre	EG	40	71.7250	37.68832	5.95905
	CG	39	79.1026	16.72973	2.67890

*Note* SAS (Students’ Academic Success); R (Resilience); AE (Academic Enjoyment); Pre (Pretest); Post (Posttest)

In Table 1, all the mean values are close to each other. It can be concluded that the two groups were at the same level of AS, AR, and AE; however, to wonder if any minimal difference exists or not, the table below must be looked at:

**Table 2:** Independent samples T test (Pretests of AS, AE, and AR)

		Levene’s Test for Equality of Variances		t-test for Equality of Means				
		F	Sig.	t	df	Sig. (2-tailed)	Mean Difference	Std. Error Difference
SAS Pre	Equal variances assumed	2.277	0.135	1.622	77	0.109	6.12179	3.77393
	Equal variances not assumed			1.638	50.751	0.108	6.12179	3.73839
R Pre	Equal variances assumed	0.055	0.815	-1.403	77	0.165	-7.58013	5.40157
	Equal variances not assumed			-1.402	75.862	0.165	-7.58013	5.40829
AE Pre	Equal variances assumed	37.044	0.000	-1.120	77	0.266	-7.37756	6.58994
	Equal variances not assumed			-1.129	54.089	0.264	-7.37756	6.53351

A look at the Sig. (2-tailed) column in Table 2 shows that all the p values are higher than 0.05; Therefore, the researchers concluded that no statistical differences existed between the groups before administering the treatment

After carrying out the treatment, the posttests of SAS, AE, and R were conducted to investigate the proposed hypotheses (Hypothesis 1: Modifications of the applied academic books foster academic success in an EFL setting. Hypothesis 2: Modifications of the applied academic books foster academic enjoyment in an EFL setting. Hypothesis 3: Modifications of the applied academic books foster academic resilience in an EFL setting.). What was discovered is shown in the tables that follow.

**Table 3:** Descriptive statistics (Posttests of AS, AE, and AR)

	Groups	Mean	Std. Deviation	N
SAS Post	EG	39.9250	4.01528	40
	CG	30.2308	7.91555	39
	Total	35.1392	7.89796	79
R Post	EG	115.0250	12.03518	40
	CG	91.8205	25.37391	39
	Total	103.5696	22.85612	79
AE Post	EG	89.9750	9.39036	40
	CG	70.9744	17.46498	39
	Total	80.5949	16.85498	79

The mean scores of both EG and CG on the posttest of SAS, AE, and AR can be seen in Table 3. The difference between the two groups on each variable is evident.

**Table 4:** Multivariate test table

	Effect	Value	F	Hypothesis df	Error df	Sig.	Partial Eta Squared
Intercept	Pillai’s Trace	0.989	2197.013	3.000	75.000	0.000	0.989
	Wilks’ Lambda	0.011	2197.013	3.000	75.000	0.000	0.989
	Hotelling’s Trace	87.881	2197.013	3.000	75.000	0.000	0.989
	Roy’s Largest Root	87.881	2197.013	3.000	75.000	0.000	0.989
Groups	Pillai’s Trace	0.575	33.846	3.000	75.000	0.000	0.575
	Wilks’ Lambda	0.425	33.846	3.000	75.000	0.000	0.575
	Hotelling’s Trace	1.354	33.846	3.000	75.000	0.000	0.575
	Roy’s Largest Root	1.354	33.846	3.000	75.000	0.000	0.575

Looking at the Wilks’ Lambda row in Table 4 shows that there was a statistically significant difference in SAS, AR, and AE, *F* (3, 75) = 33.846, *p* <.05; Wilk’s Λ = 0.425, partial η^2^ = 0.575.

**Table 5:** Tests of between-subjects effects

Source	Dependent Variable	Type III Sum of Squares	df	Mean Square	F	Sig.	Partial Eta Squared
Corrected Model	SAS Post	1855.770	1	1855.770	47.478	0.000	0.381
	R Post	10632.648	1	10632.648	27.187	0.000	0.261
	AE Post	7129.089	1	7129.089	36.523	0.000	0.322
Intercept	SAS Post	97190.606	1	97190.606	2486.521	0.000	0.970
	R Post	844869.661	1	844869.661	2160.238	0.000	0.966
	AE Post	511535.772	1	511535.772	2620.651	0.000	0.971
Groups	SAS Post	1855.770	1	1855.770	47.478	0.000	0.381
	R Post	10632.648	1	10632.648	27.187	0.000	0.261
	AE Post	7129.089	1	7129.089	36.523	0.000	0.322
Error	SAS Post	3009.698	77	39.087			
	R Post	30114.719	77	391.100			
	AE Post	15029.949	77	195.194			
Total	SAS Post	102412.000	79				
	R Post	888154.000	79				
	AE Post	535307.000	79				
Corrected Total	SAS Post	4865.468	78				
	R Post	40747.367	78				
	AE Post	22159.038	78				

Based on the data shown in Table 5, it is evident that the modifications implemented in the applied language materials have a statistically significant impact on the self-assessment scores (SAS) (F (1, 77) = 47.478; *p* <.05; partial η2 = 0.381), AR (F (1, 77) = 27.178; *p* <.05; partial η2 = 0.261), and AE scores (F (1, 77) = 36.253; *p* <.05; partial η2 = 0.322).

## Discussion

This investigation highlighted that textbooks assist instructors in all language classes, being adept at modifying textbooks for the classroom becomes an essential component of a teacher’s professional development. The instructors of languages are among the people who are fully aware of the possible weaknesses of textbooks since they are responsible for teaching textbooks and, as a result, they are the ones who bring the problems with teaching materials to light. According to [1, 2], instructors are the ones who may directly manipulate textbooks and ensure their efficacy; for this reason, it is strongly advised to depend on teachers’ assessments of the textbooks. Furthermore, the book “Life” by Helen Stephenson was investigated for their cognitive, communicative, and creative potential by [37] using a CLT model. They discovered that although the textbooks tried to adhere to the CLT techniques, they fell short of properly realizing students’ interpersonal, mental, and imaginative abilities. Additionally, some essential components of CLT—such as strategy directions, the use of authentic materials, and competence integration—were overlooked. Furthermore, their findings suggested that the social components of CLT had been harmed by the focus on culture in the book. According to the assertions made in [1], it is of the utmost importance to develop appropriate strategies for individual learning in CLT classrooms. Additionally, as stated by [38], the use of communication strategies in CLT classrooms has the effect of lowering tensions and increasing the level of involvement that students have with activities.

The evaluation of the books in the Life series incorporated a reflection on their weaknesses in terms of offering a comprehensive range of activities. The acquisition of the skills needed to communicate with both fluency and precision is a considerable challenge for language learners, as noted by [39], which claims that this is especially true. This challenge is made much more difficult by the fact that textbooks do not provide an adequate number of exercises that encourage fluency and correctness. The disorganized presentation of new lessons in this series continues to the degree that not only is the book “Life” by Helen Stephenson deficient in grammatical explanations, but it also demonstrates indifference towards the quantity of material offered in the taught structures of grammar [40]. In the same line of investigation, [41] asserts that one of the main concepts of communicative pedagogy is to teach skills in a manner that is integrated via the use of a variety of different modalities. As far as putting skills into activities is concerned, it would seem that the Life series books were just as lacking in this regard.

In addition, previous analysis refers to instances in which the vocabulary items or sentences that are familiarized by images do not clearly correlate with the meanings that were intended for them [6, 42, 43]. Therefore, it would seem that there is a need for adjustments to be made concerning the visual features of this series. As a result, it is of the utmost importance that the authors and designers of the book “Life” as well as the curriculum designers of Saudi Arabia’s Educational System make it a priority to adjust this series to the levels and requirements of the students. At present, teachers are strongly suggested to modify the exercises in these books and set the tone of their class activities based on the need analysis of the learners, multiple intelligences, as well as their taste in applying technology in their language classes. In the Prospect series, the localized series books in Iran, [10] concluded that the activities were insufficient to address the emotional aspects of learning and to implement lessons based on emotional intelligence. Students were unable to share ideas, develop a strategy for learning, comprehend their own and others’ emotions, feel fulfilled, and so on via the activities in either the main book or the workbook. Therefore, to cocomplete the academic goals, it is recommended that adjustments and additional resources be used.

Based on the findings of this research, modifications in coursebooks could enhance language growth, AE, as well as resilience. Considering that the nature of class activities is associated with increased resilience that fosters well-being [3, 44], it is plausible that individuals who hold the belief that language proficiency can be enhanced through diligent effort would maintain their resilience when confronted with obstacles. This would prevent them from going through negative emotions and enable them to maintain emotional equilibrium even in challenging circumstances. Consequently, this increased resilience may result in heightened engagement and dedication towards attaining these objectives. The results further validate the conclusions of prior studies about the associations between the ideal L2 self and both negative and good affect [45, 46].

Making the classroom a friendly and interesting environment for students should take up most of the work, as shown by the study’s findings, which demonstrated that at the end of the term, the assignments significantly enhanced students’ perceptions of classroom activities. These pre-planned activities can help teachers foster a more student-centered classroom environment by encouraging students to work together by asking each other questions, discussing their thoughts and feelings about the images, the weather, etc., and selecting activities that best fit their unique learning preferences. Students’ participation in the classroom is thus increased by putting into practice the activities that allow learners to make their voices heard. It might be beneficial to include humorous exercises within the lecture to make the lesson more enjoyable. Funny photos are another great way to liven up class. The students quickly became bored in class since the major book (Life) lacked appealing and eye-catching illustrations. The developed supplemental resources are enhanced with some endearing and humorous images. Playing games in class is another great approach to keep students interested and having fun. It is possible for students to invest a significant amount of time without really gaining any knowledge [47–49].

The results of the present research also indicated that students’ language competency may be significantly enhanced by the planned activities. Students whose learning process included the tasks finished the term with higher results than their counterparts in the control group. By completing assignments, students may improve their language skills and communication abilities. The tasks that demand that students use real-world examples are genuine, which is a key component that might enhance language acquisition. Using many real-world activities in the classroom is one teaching strategy that may be utilized to increase communication. As a result, there is a greater chance that students will become proficient in the target language, and learning takes place in a more relevant setting [7, 8]. Researchers achieved this goal by constructing assignments that prompted students to make something relevant to their everyday lives.

The hypothesis stating that the level of enjoyment experienced in academic activities has a notable and beneficial impact on the accomplishment in EFL was also verified. It should be highlighted that the learners’ emotional and cognitive states were affected by the creative activities. Students’ academic motivation and engagement increase and their academic success increases when they experience less anxiety and anger. It may be deduced that language instructors must stimulate their students’ self-awareness and self-evaluation by developing tasks and activities that foster a feeling of agency, mastery, and self-confidence.

Taking into account the third research hypothesis (Modifications of the applied academic books foster academic resilience in an EFL setting.), the outcomes witnessed the fostering influence of academic materials’ modifications on AR. That is to say, the rate of progress in AR was decided by the amount of participation in class choirs that was a result of creative activities. Book Modifications that are based on the requirements of the learners may enhance the possibility of obtaining advantages from the activity. This is because it forces the learners to think about their psychological experiences and come up with imaginative solutions to the stress that would result from the imminent study of a foreign language.

Following the self-determination assumptions [13], resilient students enjoy enhancements in their levels of motivation, contentment, and participation in the classroom. They have the capacity to recover quickly from any setbacks and become skilled at coming up with inventive solutions to any issues that arise. How they do this by establishing attainable goals and making a genuine effort to adhere to the cultural norms and social expectations of the communities in which they live. The findings of this study are consistent with the findings of [50–52], who discovered that it is beneficial for EFL students to maintain a balance in academic emotion regulation and self-awareness to effectively manage test anxiety.

### Conclusion and suggestions for further research

The analysis conducted in this study shows how useful supplemental classroom activities can be. More specifically, how language instructors adapt books in a way that makes them more practical makes them speak louder (Actions speak louder than words). Teachers should assess their students’ interests and requirements in class and then give a range of activities designed to assist them reach their objectives. Furthermore, material designers may be able to use the results of the current inquiry into localized or universal books to create academic materials that are better suited to the purported communicative features.

Similar to empirical investigations, the results of this study should be analyzed with certain limitations. The present research used a quasi-experimental approach, and intact groups were included in the sampling operations. Future research is suggested to use other methodologies to complement the findings of the present study. Moreover, the sample sizes of the EG and CG were quite small, which might impact the generalizability of the findings. Further research with more people is necessary in the future. This study does not collect the retrospective perspectives of both teachers and students on the alleged communicative ideas that are behind the book “Life”. An investigation of this kind would not only be illuminating and informative, but it would also help provide a more comprehensive picture of the present situation of the book “Life” in schools. Furthermore, the subsequent research may examine the effects of various teaching platforms and technologies on language subskills. Further investigations might investigate language competence levels, similar to the emphasis on lower/upper-intermediate learners. This study had no female participants. Future research on the inclusion of both genders could be undertaken. In addition, it is possible to investigate other significant psychological qualities, such as motivation, anxiety, openness to communication, and efficacy. Last but not least, the critical thinking and metacognitive capacities of students should be the primary emphasis of academic research in the future.

### Electronic supplementary material

Below is the link to the electronic supplementary material.

Supplementary Material 1

## Data Availability

The dataset of the present study is available upon request from the corresponding author.

## Abbreviations

EFL: English as a Foreign Language
MANOVA: The Multivariate Analysis of Variance
ELT: English Language Teaching
CLT: Communicative Language Teaching
AR: Academic Resilience
AE: Academic Enjoyment
OQPT: The Oxford Quick Placement Test
FLES: The Foreign Language Enjoyment Scale
ARS: The Academic Resilience Scale
